# Are New Generations of Female College-Student Populations Meeting Calcium Requirements: Comparison of American and Croatian Female Students

**DOI:** 10.3390/nu2060599

**Published:** 2010-05-28

**Authors:** Crystal C. Douglas, Ivana Rumbak, Irena Colić Barić, Marinela Kovačina, Martina Piasek, Jasminka Z. Ilich

**Affiliations:** 1 Nutrition, Food and Exercise Sciences, College of Human Sciences, Florida State University, Tallahassee, Florida 32306-1493, USA; Email: cacdouglas4@netscape.net (C.C.D.);; 2 Department of Food Quality Control, Faculty of Food Technology and Biotechnology, University of Zagreb, 10 000 Zagreb, Croatia; Email: icecic@pbf.hr (I.R.); icolic@pbf.hr (I.C.B.); 3 Analytical Toxicology and Mineral Metabolism Unit, Institute for Medical Research and Occupational Health, 10 000 Zagreb, Croatia; Email: marinelak@inet.hr (M.K); mpiasek@imi.hr (M.P.)

**Keywords:** BMI, dairy, food frequency questionnaire, multivitamin/mineral supplements

## Abstract

We compared calcium (Ca) sources and intake, as well as multivitamin/mineral supplement use between female students with nutrition/health background and those from general-student-populations. 314 participants 18–37 y, including 57 African-Americans and 54 Caucasian-Americans recruited from Nutrition and/or other Health Sciences departments (NHS), and 100 African-American and 103 Croatian women representing general-student-population (GSP), completed food frequency questionnaire assessing their usual Ca intake and supplement use. NHS populations met recommendations and consumed significantly more Ca, particularly from dairy sources, and were more likely to take supplements than GSP groups, suggesting that health education may influence Ca intake.

## 1. Introduction

Under the guidelines provided by the Institute of Medicine of the National Academy of Sciences in 1997, known as Dietary Reference Intakes (DRI) [1], the new recommendation for calcium (Ca) for women aged 19–50 in the US is 1,000 mg/day. Although Ca consumption in the United States varies according to demographics including gender, race, ethnicity, and region of the country, this recommendation has been rarely met, at least based on the previous surveys. According to the USDA's 1987-88 Nationwide Food Consumption Survey (87-88 NFCS), females, African Americans, and those residing in the South, consume the least amount of Ca [2]. Additional studies suggest that women in the United States, particularly African Americans, do not consume adequate amounts of dairy productand, as a result, are not meeting the recommendations for dietary Ca [2,3]. In fact, data from the Continuing Survey of Food Intakes by Individuals 1994-1996, 1998 (CSFII), report the median usual Ca intake for non-African American and African American women aged 19–30 at approximately 675 mg/day and 500 mg/day, respectively [4]. Barriers affecting Ca intake in women include lack of knowledge regarding dietary sources and importance of Ca for bone health [5,6], while taste preferences, and lactose maldigestion symptoms are commonly reported as barriers among African American women [6]. 

Calcium intake in Croatia, a country located in south-central Europe, is also driven by demographics such that females and those residing in rural areas consumed the least amount [7]. Croatia follows the recommendations of 800 mg/day for dietary Ca [8]. However, most of the scientific reports from Croatia compare Ca intake with reference to the DRIs [9,10]. A recent study conducted in Croatia reported an average Ca intake of 1,444 mg/day among adolescent and adult females [10], while an older one reported a mean Ca intake of 900 mg/day for Croatian women, with 63% of the women falling short of the DRI’s levels of 1,000 mg per day [11]. It is of interest to note that one of the first groundbreaking studies demonstrating the significance of dietary Ca on bone mass and fracture rates was conducted in Croatia [12]. 

In the last decade, great public efforts have been made toward educating population about the relevance of dietary Ca for bone and other health conditions with the hope of raising the intake closer to the recommended level. College students, although in general known for bad eating habits, poor nutrition and low intake of some crucial nutrients including Ca [13], do have a greater comprehension for public-health education messages and therefore greater chances of benefiting from them. Moreover, students who are in health science disciplines acquire knowledge about nutrition but it is questionable whether they implement that knowledge toward their own dietary practices. In view of the global increase in documented cases of osteoporosis, we were interested in reassessing Ca intake among new generations of various American as well as Croatian female college-student populations. 

Therefore, the aim of this study was to assess and compare current Ca intake as well as the dietary sources of Ca between African American and Caucasian American female college students enrolled in nutrition and/or health related courses and African American and Caucasian Croatian students recruited from the general student population to identify whether a background in health education negates demographic influences reported to affect dietary Ca consumption. Additionally, we compared multivitamin/mineral use between students enrolled in nutrition and/or health related courses and students representing the general population. To the best of our knowledge, this is the first study where dietary Ca intake and multivitamin/mineral use have been examined among students with a nutrition/health background and compared with the general student population in the country and abroad. 

## 2. Subjects and Methods

### 2.1. Subjects

Participants included 314 female college students aged 18–37 y, recruited from several college campuses. Fifty-seven African American and 54 Caucasian American women were recruited from Nutrition and/or other Health Sciences departments (Nutrition-Health Students; NHS) from Florida State University. African American students (n = 100) from the University of Alabama and Croatian (Caucasian) women (n = 103) from the University of Zagreb were recruited from the general student population (General-Student Population; GSP) from various departments. All participants considered themselves healthy and free of chronic disease. About 90% were undergraduates and none of the students was participating in the college sports team or was engaged in strenuous physical activity. Study protocol was approved by the Institutional Review Board of each university. After the study was explained in the classroom or on an individual basis, an informed consent was signed by each participant. 

### 2.2. Dietary Intake

Dietary Ca intake was assessed by a food frequency questionnaire (FFQ), which included 52 foods and beverages recognized for their Ca content, as described previously [14]. The questionnaire was slightly modified for the American students to include foods typically consumed in the southern region that are rich in Ca, like okra and collard greens. Likewise, the FFQ for Croatian students included foods typical for that area, but comparable to the American items. They were also presented with the photographs of foods indicating small, medium and large portions, as Croatian students are not accustomed to serving sizes expressed in cups and ounces. All participants were instructed how to complete the questionnaire and were encouraged to describe the frequency of consumption by reporting for the day, week, and/or month, and to describe the amounts consumed using weight, volume, and/or serving sizes. Desserts were divided into two categories: dairy desserts included foods rich in Ca such as ice cream, pudding, and cheesecake and those including non-dairy desserts such as brownies, candy bars, or cookies. Dietary Ca content was calculated from the USDA data base [15] for the American students and from the Croatian Food Tables [16] for the Croatian students. 

Consumption of multivitamin/mineral or individual Ca supplements (including antacids), with brand names, quantity and frequency was recorded for all participants. The brand names for the African American GSP population were not available, therefore, upon the confirmation of supplement intake, a Ca value of 165 mg was assigned (the average Ca content found within multivitamins/minerals) [17]. Consumption of tap and bottled water, including brand names and quantity, was reported for all participants, except for the African American GSP. Croatian participants also reported the consumption of mineral waters, a popular and common drink in that area [18]. Ca from tap water was calculated from published information about Ca content in tap water of the respective countries/states and that from bottled and mineral waters was derived from the label declarations. The amount of Ca is expressed in mg/day as dietary (from food only) and total (including food, supplements, and water). Height and body weight were self-reported and body mass index (BMI; kg/m^2^) was calculated. 

### 2.3. Statistical Analyses

All data are presented as means ± standard deviation, unless noted otherwise and analyses were performed using SPSS, version 15.0 for Windows. Assumptions for normality were verified by the Kolmogorov-Smirnov test and found satisfactory. Differences between the four populations regarding the means of continuous variables were tested using one-way ANOVA and post-hoc multiple comparisons (Scheffé) to account for multiple groups and differences in sample sizes. All participants, as well as those within each population were divided into groups below and above the recommended Ca intake (1,000 mg/day) [1]. Ca intake per kg of body weight in each group of participants was calculated as well. The accepted level of statistical significance was at *p* < 0.05. 

## 3. Results

Table 1 shows descriptive characteristics, Ca intake, and sources of dietary Ca for the populations. The mean intake of dietary Ca from food was highest among the African American and Caucasian American NHS populations; 63% of the African American and 57% of the Caucasian American participants met or exceeded the daily recommended intake for Ca of 1,000 mg/day [1]. The lowest reported dietary Ca intake from food among the African American and Caucasian American NHS students was 465 mg/day and 424 mg/day, respectively. On the contrary, only 24% of the African American and 30% of the Croatian GSP participants met or exceeded the Ca requirements. Likewise, the lowest reported dietary Ca intake from food was observed within the GSP; 111 mg/day for the African Americans and 321 mg/day for the Croatians. 

**Figure 1:**
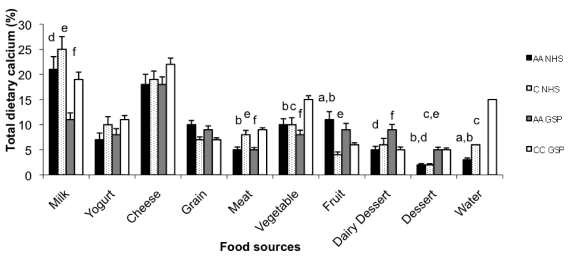
Percentage of total dietary calcium (X ± SE) from various food sources among the Nutrition-Health Students (NHS) and General-Student Populations (GSP). AA = African Americans, C = Caucasian American, CC = Caucasian Croatian. Water includes mineral water for Croatian population only; ^a^African American NHS differ significantly from Caucasian American NHS,^ b^African American NHS differ significantly from Croatian students, ^c^Caucasian American NHS differ significantly from Croatian students, ^d^African American GSP students differ significantly from African American NHS, ^e^African American GSP students differ significantly from Caucasian American NHS, ^f^African American GSP students differ significantly from Croatian students.

Dairy products provided more than 50% of dietary Ca among the NHS Caucasian American students and the Croatians (Figure 1). Milk was the largest contributor of Ca in the African American and Caucasian American NHS populations, while cheese was the largest contributor of Ca in the GSP students. About 19% of African American and 17% of Caucasian American NHS participants, and 32% African American and 13% Croatian GSP students did not report any milk consumption. 33% of African American and 30% of Caucasian American NHS and 45% of African American and 11% of Croatian GSP participants did not report any yogurt consumption. Desserts (including dairy based) provided 14% of dietary Ca consumption within the African American GSP students, the highest among all student populations. 

**Table 1:** Descriptive characteristics (Mean ± SD), calcium intake (mg/day), servings per day and sources of dietary calcium among the Nutrition-Health Students (NHS) and General-Student Populations (GSP).

Variables	NHS				GSP				
	African American (n = 57)		Caucasian American (n = 54)			African American (n = 100)		Croatian (n = 103)	
Age (y)	21.7 ± 3.6		21.2 ± 2.2			20.9 ± 1.7^f^		22.0 ± 2.0	
Height (cm)	163.9 ± 6.1^b^		165.4 ± 5.2^c^			164.1 ± 6.61^f^		169.0 ± 5.7	
Weight (kg)	71.1 ± 16.6^a,b^		63.3 ± 11.1^e^			70.6 ± 14.2^f^		60.4 ± 6.8	
BMI (kg/m^2^)	26.5 ± 6.3^a,b^		23.1 ± 4.0^e^			26.2 ± 5.3^f^		21.1 ± 2.0	
Calcium from food	1398 ± 854^b,d^		1273 ± 842^c,e^			818 ± 501		889 ± 371	
		**#**		**#**			**#**		**#**
Cheese	268 ± 302^d^	1.3	257 ± 314	1.3		159 ± 190	0.8	201 ± 175	1.0
Milk	312 ± 408^b,d^	1.0	313 ± 272^c,e^	1.0		101 ± 160	0.3	191 ± 197	0.6
Yogurt	105 ± 179	0.3	129 ± 184^e^	0.4		68 ± 96	0.2	99 ± 70	0.3
Fruit /juices	156 ± 204^a,b^	2.2	55 ± 88	0.8		93 ± 190	1.2	51 ± 32	0.7
Bread/grains	126 ± 129^a,b,d^	3.6	75 ± 58	2.1		64 ± 50	1.8	58 ± 29	1.7
Meat/fish/beans	67 ± 63	1.3	101 ± 130^e^	2.0		38 ± 33^f^	0.8	73 ± 30	1.5
Vegetables	138 ± 168^d^	1.4	129 ± 195^e^	1.3		64 ± 72^f^	0.6	130 ± 74	1.3
Dairy dessert*	59 ± 74	0.7	77 ± 136	1.0		73 ± 89	0.9	43 ± 60	0.5
Dessert/cookies	24 ± 32^b^	2.4	21 ± 30^c,e^	2.1		38 ± 41	3.8	42 ± 39	4.2
Vitamin/miner supplements	87 ± 171^d^		68 ± 131			36 ± 69		None	
Water**	35 ± 34^a,b^		63 ± 53^c^			N/A		151 ± 71	
Total calcium	1519 ± 873^b,d^		1404 ± 853^c,e^			855 ± 501		1040 ± 391	

# Refers to number of servings

* Includes ice cream, pudding, frozen yogurt, and custard

** Not collected from the African American GSP students. Water includes mineral water consumption (23.9 ± 32.6 mg/Ca) for the Croatian population only.

Values with different subscripts indicate significant differences (p < 0.05; Scheffé test following ANOVA)

^a^African American NHS differ significantly from Caucasian American NHS

^b^African American NHS differ significantly from Croatian students

^c^Caucasian American NHS differ significantly from Croatian students

^d^African American GSP students differ significantly from African American NHS

^e^African American GSP students differ significantly from Caucasian American NHS

^f^African American GSP students differ significantly from Croatian students

**Figure 2:**
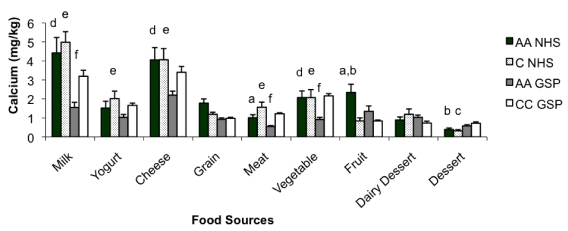
Intake of calcium, mg/kg body weight, (X ± SE) from various food sources among the Nutrition-Health Students (NHS) and General-Student Populations (GSP). AA = African Americans, C = Caucasian American, CC = Caucasian Croatian, ^a^African American NHS differ significantly from Caucasian American NHS, ^b^African American NHS differ significantly from Croatian students, ^c^Caucasian American NHS differ significantly from Croatian students, ^d^African American GSP students differ significantly from African American NHS, ^e^African American GSP students differ significantly from Caucasian American NHS, ^f^African American GSP students differ significantly from Croatian students.

Ca intake analyzed per kg of body weight is presented in Figure 2. The African American and Caucasian American NHS populations were consuming the most Ca from food per kg of body weight (20.6 ± 14.2 and 20.1 ± 11.8, respectively). Although no significant difference in Ca intake (mg/kg body weight) was noted between the GSP African American and Croatian students (11.9 ± 7.6 and 14.9 ± 6.6, respectively; *p* = 0.186), both groups consumed significantly less Ca than the NHS African American and Caucasian American students (*p* < 0.018). Specifically, the NHS students were consuming significantly more Ca from milk, cheese, and yogurt (*p* < 0.05). 

Among the NHS students, 30% of the African Americans and 28% of the Caucasian Americans were taking multivitamins/minerals, which increased the total average Ca intake for each population by 87.2 ± 170.9 mg/day and 67.7 ± 131.4 mg/day, respectively. 22% of the African American GSP participants reported taking multivitamin/mineral supplements which increased their average Ca intake by 36.3 ± 68.7 mg/day. Croatian students were not taking multivitamin/mineral supplements. Water consumption was reported for all populations except the African American students representing the general student population. For the Croatian students only, Ca intake from tap and mineral water provided a significant percentage (15%, mostly coming from mineral water) of their total daily Ca intake. The contribution of Ca from water to total Ca intake was trivial for all African and Caucasian American students. They did not consume mineral water. No significant difference in total Ca intake was noted between the African American and Caucasian American NHS students (1519.2 ± 872.6 *vs.* 1403.8 ± 852.6, respectively) yet both the NHS groups consumed significantly more Ca than either one of the GSP groups (*p* < 0.05).

## 4. Discussion

Our data show that Ca intake was the highest in the NHS populations, with 63% of the African American and 57% of the Caucasian American participants consuming a level of Ca at or above the DRIs, while only 24% of the African American and 30% of the Croatian GSP participants met or exceeded Ca requirement. We also demonstrated that Ca intake was not driven by race but by the influence of health education. To our surprise, mean Ca intake and consumption of Ca from dairy foods within the NHS and GSP groups was similar between races. A recent study, analyzing duplicate 24-h recalls from African American and non-African American women, reported that African American women consumed less dairy and had a lower intake of calcium than their non-African American counterparts [4]. Ca intake among this Croatian population was in agreement with a previous study, reporting a mean Ca intake of 900 mg/day based on a FFQ [11]. Our data indicate that students enrolled in nutrition and/or health related courses consumed significantly more Ca, specifically Ca from dairy sources, and were more likely to take a multivitamin/mineral supplements than those from the general student population. These findings suggest that health education can potentially nullify demographic influences reported to affect Ca consumption, including race, ethnicity, and region of the country. 

According to the Continuing Survey of Food Intakes of Individuals (CSFII 1994-96), 78% of American women aged 20 and older are not meeting their recommended intake for Ca [19]. Additionally, Ca and dairy intake is typically poorer in African Americans as opposed to Caucasians [2,3,4]. However, this racial disparity was not evident among either the NHS or GSP populations. Moreover, average Ca intake (both dietary and total) exceeded the recommended level of 1,000 mg per day for both the African American and Caucasian NHS populations. Dietary Ca intake was similar but fell short of the daily requirements for both GSP populations. However, Croatians reached the recommended level with total Ca intake, mostly due to the mineral water consumption. Calcium intake for this Croatian population was substantially lower than an earlier report, also based on a FFQ, which reported a mean intake of approximately 1,500 mg among Croatian female university students [11]. The reported differences could be attributed to the different FFQ tools and geographical differences as the previous study included college students from 5 campuses throughout Croatia. 

The African American GSP students reported a Ca intake below the recommended 1,000 mg/day, and of all the groups, consumed the least amount of Ca from dairy products (37%) and the most from desserts (14%). This finding is in agreement with data from the Continuing Survey of Food Intakes by Individuals 1994–1996, 1998 (CSFII), and the National Health and Nutrition Examination Survey 1999–2000 (NHANES), which found that African Americans consume less dairy products per day than their non-African American counterparts [4]. Additionally, this finding suggests that the primary food sources of Ca within this population are poor sources of Ca and relatively high in energy and fat. A previous study in the United States showed that African Americans tend to consume more whole-fat dairy products, including cheese, than their non-African American counterparts [20]. 

Lower Ca intake among the African American GSP students was somewhat anticipated as the true (or perceived) prevalence of lactose maldigestion in the United States is reported to be approximately 15% for Caucasians and nearly 80% for African Americans [21]. This condition, although grossly misdiagnosed, often leads to the avoidance of dairy products even though studies have demonstrated that symptoms can be lessened with a gradual introduction of dairy products [22,23,24]. Nevertheless, Ca intake among the African American NHS students was nearly two-fold higher than Ca intake among the African American GSP students, confirming the newer findings that prevalence of lactose maldigestion is overestimated [25]. These findings also suggest that nutrition/health education is a powerful tool with the potential to influence dietary intake and dismiss dietary misconceptions. 

Cheese and milk were the largest contributors to Ca for all populations. In both the United States and Croatia, more than 50% of dietary Ca was consumed via dairy products [2,11]. However, milk and yogurt avoidance was highest among the African American GSP students, followed by the African American NHS students. This might be a problem as dairy products are the best sources of Ca, and in addition to Ca, they also provide magnesium, phosphorus, some B vitamins and vitamin D, as well as protein. Dairy intake has been positively associated with greater intakes of these nutrients [3,4]. 

After Ca intake was adjusted for body weight, we found a striking difference between the populations. The NHS populations were consuming a similar amount of Ca from food per kg of body weight, and significantly more than either of the GSP populations. Findings for the Croatian students are consistent with an earlier report, which documented a Ca intake of 14.5 mg/kg [11]. These data, adjusted for body weight, suggest again that students with a nutrition/health background consume a diet richer in Ca and dairy products than their counterparts from the general student-populations.

Regular multivitamin/mineral use among the NHS populations was similar to that reported in the study conducted among US female medical students (30%) [26]. In that study, students more likely to use multivitamins exercised regularly, had children, were underweight (BMI < 18.5), and consumed little to no alcohol. We did not gather these data but it is encouraging that multivitamin/mineral use was higher among the populations with a nutrition/health background than the GSP populations, as a possible means of correcting for dietary deficiencies. The absence of multivitamin/mineral use among the Croatian population may be partly due to the higher costs and/or limited availability of multivitamins in Croatia as well as the different cultural outlook on supplement use [18]. 

The contribution of Ca provided by water was miniscule, but that from the mineral waters was significant for the Croatian population. This finding was expected as mineral water is a naturally rich source of Ca and a popular beverage in Croatia [18]. Furthermore, a study analyzing the contribution of mineral water to dietary Ca intake in French women aged 35–60 reported that women drinking mineral water consumed significantly more Ca than those drinking tap water, and that mineral water itself may supply up to 25% of total daily Ca [27]. 

There are some limitations of our study. Dietary data were self-reported and therefore, Ca and multivitamin/mineral intake could have been biased if students thought these behaviors would be viewed as healthier. Dietary intake was assessed one time using a FFQ designed to assess Ca, which might not represent a complete dietary history, account for changes in dietary intake occurring seasonally, or evaluate dietary consumption with regards to total energy intake. However, various types of FFQ are often used in large studies because they are easy to administer and require only a brief commitment from the participant. If the tool is administered correctly, the analyses provide quantitative data reflecting usual dietary intake, particularly for Ca that has a tendency to be relatively stable [28]. Finally, we did not obtain total energy intake which would have provided us with data regarding the proportion of energy obtained from foods rich in Ca. 

To our knowledge, this is the first report of dietary Ca intake, including multivitamin/mineral use and water among general-student populations and those with a nutrition/health background. Additionally, the inclusion of 4 distinct student populations, including students from Croatia, made our comparisons more comprehensive. The significant difference in Ca intake between the students recruited from the Nutrition or Health Sciences departments and those recruited from the general-student population suggest that some degree of beneficial healthy behavior, including the importance of Ca in the diet, is taking place within the NHS classroom. The relevancy of our findings is critical as personal health practices, including dietary practices, among health professionals have been linked to their own patient counseling. Students currently enrolled in nutrition or health sciences courses do represent the future of our medical field and thus, emphasis on health-promotion education is important if these learned healthy practices will one day translate to healthy behavioral counseling. This message is valuable as Ca intake has not only been associated with the prevention and treatment of osteoporosis [29], but hypertension [30], insulin resistance syndrome [20], and certain cancers [31,32]. Furthermore, education in the classroom may translate to major savings in the future as a recent report suggests that if Americans increased their dietary Ca (dairy) intake as part of a healthy diet, and met the current recommendations, we would observe a decrease in the prevalence of many conditions and diseases, which would translate to a major reduction in healthcare costs [33]. 

## 5. Conclusions

Our data indicate that more than half of the students with nutrition/health sciences background consumed Ca at or above the recommended level of 1,000 mg/day whereas less than one third of the general student population met or exceeded this requirement. African American and Caucasian NHS populations consumed significantly more Ca, specifically from dairy sources, and were more likely to take multivitamin/mineral supplements than either GSP group, suggesting that health education may influence Ca intake. 
